# Characteristics of a Temperature-Sensitive Mutant Strain of *Salmonella* Enteritidis and Its Potential as a Live Vaccine Candidate

**DOI:** 10.3390/vetsci10050313

**Published:** 2023-04-25

**Authors:** Hyunjin Shin, Tae-Min La, Hong-Jae Lee, Taesoo Kim, Seung-un Song, Gyu-Hyung Park, In-Soo Choi, Seung-Yong Park, Joong-Bok Lee, Sang-Won Lee

**Affiliations:** College of Veterinary Medicine, Konkuk University, Seoul 05029, Republic of Korea

**Keywords:** chickens, live-attenuated vaccine, safety, *Salmonella* Enteritidis, single nucleotide polymorphism, temperature-sensitive

## Abstract

**Simple Summary:**

*Salmonella enterica* serovar Enteritidis can cause human salmonellosis; however, its main carrier, poultry, does not show any clinical symptoms. In this study, we evaluated the potential of a mutant *Salmonella* Enteritidis strain as a vaccine candidate. Compared to the wild-type *Salmonella* Enteritidis, the vaccine candidate *Salmonella* Enteritidis strain was more attenuated. Moreover, single nucleotide polymorphism analysis revealed that genetic changes in the vaccine candidate strain corresponded with its phenotypes. Thus, the *Salmonella* Enteritidis strain constructed in our laboratory might be valuable in developing a novel live-attenuated vaccine against *Salmonella* Enteritidis.

**Abstract:**

*Salmonella* Enteritidis is a common foodborne pathogen transmitted through poultry products, which are its main carriers. Poultry are vaccinated against *Salmonella* Enteritidis in many countries, despite the absence of clinical symptoms, using commercially available live-attenuated vaccines. We previously constructed a highly attenuated temperature-sensitive (ts) *Salmonella* Enteritidis mutant, 2S-G10. In the present study, we describe the construction and attenuation-associated characteristics of 2S-G10. We infected 1-day-old chicks with 2S-G10 and the parental strains to evaluate the attenuation. One week after infection, 2S-G10 was not detected in the liver, cecum, or cecal tonsil tissues of the orally inoculated chicks, contrary to the parental strain. This indicates that 2S-G10 was highly attenuated when compared to the parental stain. In vitro experiments revealed the inability of 2S-G10 to grow at the normal body temperature of chickens and invade chicken liver epithelial cells. Moreover, single nucleotide polymorphism (SNP) analysis between the complete genome sequence of 2S-G10 and its parental strain revealed SNPs in *bcsE*, *recG*, *rfaF*, and *pepD*_1 genes, which are involved in epithelial cell invasion and persistence in host systems, growth, lipopolysaccharide core biosynthesis, and cellular survival under heat stress, respectively. These potential characteristics are consistent with the findings of in vitro experiments. Conclusively, chemical treatment–induced random genetic mutations highly attenuated 2S-G10, implying its potential to be developed as a novel live-attenuated vaccine against *Salmonella* Enteritidis.

## 1. Introduction

*Salmonella enterica* serovar Enteritidis (*S.* Enteritidis) is one of the most common *Salmonella* serotypes causing foodborne infections in humans worldwide [1]. Human salmonellosis is frequently caused by the consumption of *Salmonella*-contaminated foods, particularly poultry products and eggs [1,2]. Since poultry products and eggs have been identified as major sources of *S*. Enteritidis in food consumed by humans worldwide, there is an urgent need for efficient programs to control *Salmonella* infections in poultry [3]. The currently available commercial vaccines for poultry are based on live-attenuated *S*. Enteritidis strains, such as AviPro *Salmonella* Vac E (Lohmann Animal Health, Cuxhaven, Germany) and Salmovac SE (IDT Biologika, Dessau-Rosslau, Germany). The AviPro *Salmonella* Vac E is a metabolic drift mutant strain produced by chemical mutagenesis from a wild-type *S*. Enteritidis strain [4]. The Salmovac SE contains the double-attenuated adenine-histidine auxotrophic *S*. Enteritidis strain (https://www.thepoultrysite.com/focus/ceva/ceva-salmovac, accessed on 24 February 2022). Live attenuated vaccines can induce sufficient immune responses to protect chickens against *Salmonella* infection; however, live attenuated strains still have safety issues and pose risks of long-term circulation of the vaccine strain within the immunized flock [5,6]. Therefore, to produce *S*. Enteritidis-free poultry products, a sufficiently attenuated non-pathogenic vaccine strain should be developed.

Given that wild-type *Salmonella* strains typically thrive at 42 °C, which is the normal body temperature of chickens, it is crucial to reduce colonization at this temperature. The *Mycoplasma gallisepticum* vaccine strain ts-11, a temperature-sensitive (ts) mutant generated by treating a virulent Australian field isolate with *N*-methyl-*N*′-nitro-*N*-nitrosoguanidine (NTG), is commercially used in the chicken industry worldwide [7,8]. NTG is a chemical mutagen that induces a wide spectrum of random mutations through the methylation of nucleic acids [9,10]. NTG primarily induces G/C to A/T transitions, followed by A/T to G/C transitions and A/T to C/G transversions [11]. We previously developed an NTG-treated ts mutant strain of *S.* Enteritidis, 2S-G10, as a potential vaccine candidate and assessed its immune responses and protective efficacy; prime and booster vaccinations via oral and intramuscular routes could induce sufficient humoral and cellular immune responses to protect chickens from challenge infection [12].

The present study aims to characterize the phenotypic features and genetic changes responsible for the attenuation of the ts mutant strain. In addition, we describe the construction method of the ts mutant strain 2S-G10 by treating wild-type strains with NTG.

## 2. Materials and Methods

### 2.1. Construction of ts Mutant 2S-G10 Strain

The ts mutant strain was constructed by exposing a culture of *S*. Enteritidis 6NB strain isolated from chicken livers to 1 mg/mL NTG using a previously described method with modifications [13]. Briefly, 1 mg/mL NTG solution was prepared with phosphate-buffered saline (PBS; Gibco, Paisley, UK) and sterilized using filtration. The wild-type 6NB strain was incubated with shaking in 10 mL tryptic soy broth (TSB; BD, Sparks, MD, USA) at 37 °C for 4 h. The pellet was harvested via centrifugation at 16,200× *g* for 5 min and washed thrice with PBS. After the final wash, 300 μL of the suspension was inoculated into 4.2 mL of TSB. A total of 500 microliters of the NTG solution was added to the suspension and mixed thoroughly. After incubation at 37 °C for 1–3 h, the solution was centrifuged at 16,200× *g* for 5 min and washed thrice with PBS. The washed pellets were suspended in 5 mL TSB, and the suspension was serially ten-fold diluted. Each dilution was placed on ChromoSelect agar (Sigma-Aldrich, St. Louis, MO, USA). After incubation for 18 h at 33 °C, well-separated single colonies were picked from the agar plate and inoculated individually into 1 mL of TSB. After incubation for 18 h at 33 °C, the inocula were serially ten-fold diluted. Each dilution was placed on two 96-well plates at a 10^0^–10^−9^ dilution and incubated at 42 °C and 33 °C, respectively, to detect the presence of mutants capable of enhanced growth at the non-permissive temperature. The selected mutant strains were sub-cultured at least three times on xylose lysine deoxycholate agar (BD, Sparks, MD, USA).

### 2.2. Attenuation Evaluation of the Mutant Strain

Thirty-eight 1-day-old specific pathogen–free white leghorn chicks were randomly divided into three groups and housed separately in isolators. Commercial feed and drinking water were provided *ad libitum*. Cleaning and feeding regimes were organized throughout the trial to prevent cross-contamination. After the adaptation period, the chicks were orally administered 100 μL of the inocula at 1 × 10^7^ CFU/mL; each group was administered a culture of *S*. Enteritidis mutant strain, a culture of *S*. Enteritidis parental strain, or 100 μL PBS (negative control). On day 7 post-inoculation, all chicks from each group were sacrificed to determine *Salmonella* colonization and persistence in the liver, cecum, and cecal tonsils. The tissues were pre-enriched in buffered peptone water (BPW; BD, Sparks, MD, USA) at 37 °C for 24 h. One hundred microliters each of pre-enriched tissues were inoculated in Rappaport Vassiliadis R10 broth (RV broth; BD, Sparks, MD, USA) at 33 °C and 42 °C, respectively, for 24 h. *S*. Enteritidis-specific PCR [14] was performed using DNA extracted from the pre-enriched BPW. Bacterial genomic DNA was extracted using a commercial kit, Patho Gene-spin (iNtRON Biotechnology, Seongnam, Republic of Korea), according to the manufacturer’s instructions. After the extraction of genomic DNA, PCR was performed [15]. The primers for the PCR analysis are listed in Table 1. We confirmed that the genetic changes in the mutant strain did not influence the PCR primer binding sites. To detect viable *Salmonella*, a loop of the enriched RV broth was streaked onto ChromoSelect agar, and *Salmonella*-type colonies were examined after incubation for 20 h at 37 °C. In case of uncertainties based on this examination, the absence of *Salmonella* spp. strains in the organs was confirmed using PCR [14].

### 2.3. One-Step Growth Curve Analysis

The changes in the growth ability of the mutant strain were determined by measuring the optical density (OD) and CFUs of bacterial suspension every hour for 8 h. The bacteria were pre-cultured in TSB. The pre-cultured ts mutant strain 2S-G10 and parental strain 6NB were diluted with fresh TSB to 1 × 10^5^ CFU/mL for the measurement of OD and were diluted 1:500 with fresh TSB for the measurement of CFUs. The mutant and parental strain cultures were incubated with shaking at 33 and 42 °C. The optical densities of the strains were measured at hourly intervals at 600 nm (OD_600_) from 0 to 8 h. At the same time, the samples were subjected to the counting of viable cells on tryptic soy agar (TSA; BD, MD, USA). Counts are reported as log_10_ CFU/mL.

### 2.4. Invasion Assay

Invasion assay was performed using a previously described method with modifications [16]. Chicken hepatoma cell line LMH grown in Dulbecco′s modified Eagle′s medium (Welgene, Gyeongsan, Republic of Korea) supplemented with 10% fetal bovine serum (Gibco, NY, USA) was used. Briefly, the cells were seeded in 6-well tissue culture plates to obtain confluent monolayers on the day of infection. The overnight cultured mutant and parental strains were diluted 1:10 in fresh TSB and incubated at 33 °C and 42 °C, respectively, until they reached the log phase. Cell monolayers were washed with PBS and then infected with approximately 1 × 10^7^ CFU/mL bacterial cultures for 1 h at 37 °C. Then, the plates were washed with PBS and incubated in a culture medium containing 100 μg/mL gentamicin for 1 h at 37 °C. Thereafter, the cells were washed with PBS and lysed using cold 1% Triton X-100 (Sigma-Aldrich, MO, USA). Viable intracellular bacteria were quantified using serial dilutions plated on TSA. The results are expressed as percentages of CFU recovered relative to the number of bacteria deposited per well. Experiments were performed in triplicates for each strain.

### 2.5. Complete Genome Sequencing

For Illumina sequencing of the 2S-G10 strain, QiaAmp Mini Kit (Qiagen GmbH, Hilden, Germany) was used to extract the DNA. A TruSeq Nano DNA Sample Preparation Kit (Illumina, San Diego, CA, USA) was used for library preparation according to the manufacturer’s instructions. Illumina sequencing was performed on the Illumina NextSeq 500 platform. The parental strain was previously sequenced in our laboratory [17].

### 2.6. Comparative Genome Analysis

We conducted quality control using the fastp tool to remove the adapters and bad-quality called bases from each sequence read [18]. We then used the Burrows–Wheeler Aligner to map these reads to the parental strain [19]. The genomic sequence of the parental strain 6NB was used as the reference genome. After mapping, we cleaned up read pairing information and flags using SAMtools [20]. We also used SAMtools to generate a compressed bam-file, sort it into coordinate order, and remove duplicate reads. The flagstat results were used for further analysis. We excluded reads with low mapping quality and unmapped sequence reads.

Next, we called variants using the Freebayes tool with the reference genome scaffold file and its index and the mapping file and its index [21]. Then, we filtered the called variants that had been called with quality >30. Lastly, the filtered variants called were used as input for SnpEff to identify variants of interest and predict the potential effects of variants of genes [22]. The circular genome of the parental strain 6NB was imaged using Geneious Prime software v2022.2.1 (https://www.geneious.com, accessed on 7 July 2022). *Salmonella* pathogenicity islands (SPIs) and 63 single nucleotide polymorphisms (SNPs) in the ts mutant strain were annotated. SPIs were predicted using SPIFinder 2.0 [23].

### 2.7. Statistical Analysis

The percent invasion values of the bacterial strains in the invasion assay were compared using the Wilcoxon test. Isolation rates of *S*. Enteritidis strains in the experimental groups were compared with that of the negative control group using a two-sided Fisher’s exact test. Values were considered significantly different at *p* < 0.05. Statistical analyses were performed using R software (version 4.1.2; RStudio, Inc., Boston, MA, USA).

## 3. Results

### 3.1. Construction of the ts Mutant Strain

A total of 62 mutant strains were obtained after NTG treatment. Their temperature sensitivity was confirmed by comparing their growth rates at 33 °C and 42 °C, and the mutants that grew considerably better at 33 °C than at 42 °C were selected. As a result, the 2S-G10 strain with a two-log difference was selected.

### 3.2. Attenuation of the ts Mutant Strain

To evaluate the attenuation of the selected ts mutant even in 1-day-old chicks, we evaluated the *Salmonella* re-isolation rates in their internal organs 1 week after inoculation. As shown in Table 2, *S*. Enteritidis strain was not detected in the liver, cecum, or cecal tonsil of chicks in the ts mutant strain 2S-G10-inoculated and PBS-treated groups. In contrast, the chicks in the parental strain 6NB-inoculated group showed significantly high re-isolation rates in the liver and cecum.

### 3.3. One-Step Growth Curve Analysis

**Figure 1:**
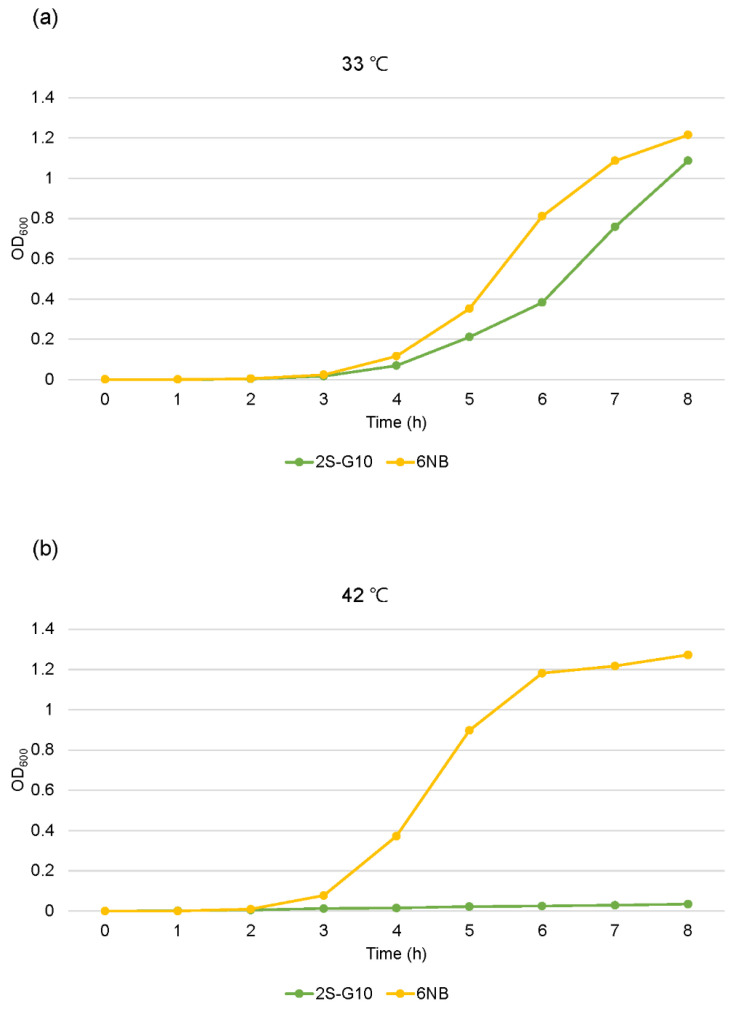
One-step growth curves based on OD measurement of the mutant and parental strain cultures. Growths of the ts mutant strain 2S-G10 and parental strain 6NB were measured every hour for 8 h at 33 °C (**a**) and 42 °C (**b**). Optical density of the samples was measured at a wavelength of 600 nm (OD_600_). The time-zero populations were set as 5.2–5.7 log_10_ CFU/mL.

**Figure 2:**
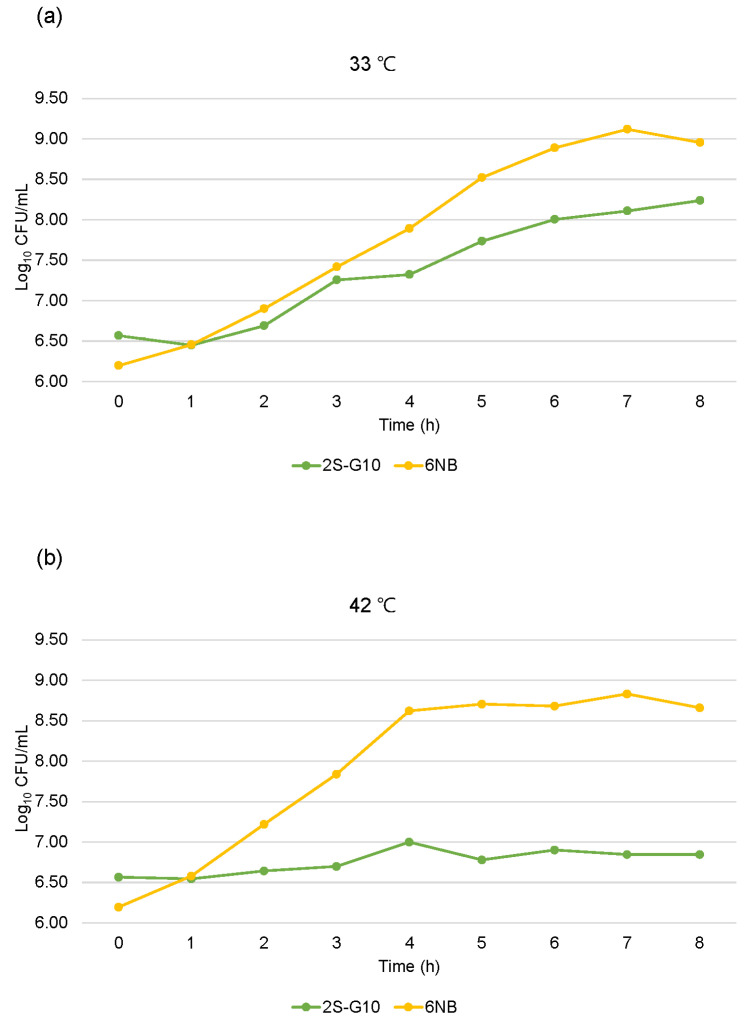
One-step growth curves based on CFU counts of the mutant and parental strains. Growth of the ts mutant 2S-G10 and parental 6NB strains was measured every hour for 8 h at 33 °C (**a**) and 42 °C (**b**) and reported as the log_10_ CFU/mL. The time-zero populations were set as 6.1–6.6 log_10_ CFU/mL.

The growth of the ts mutant strain was compared with that of the parental strain (Figure 1). Growth analysis showed that the 2S-G10 strain grew more slowly than the parental strain at 33 °C, while it could not properly multiply at 42 °C, the host body temperature (Figure 1b). Growth analysis findings based on CFU counts were consistent with the results of analysis based on the OD_600_ value (Figure 2). Both analyses revealed that the 2S-G10 strain grew more slowly than the parental strain at both 33 °C and 42 °C, with considerable growth suppression at 42 °C.

### 3.4. Invasion Assay

**Figure 3:**
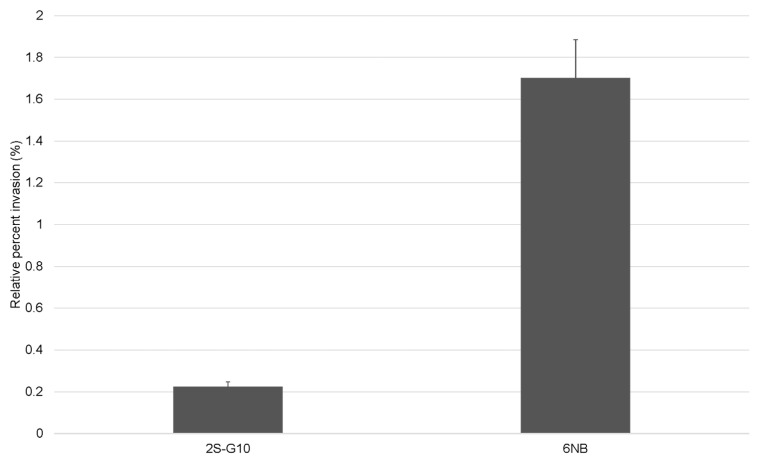
Invasion rates of the ts mutant strain 2S-G10 and parental strain 6NB into LMH cells. Values indicate the percentages of the recovered bacteria after inoculation and gentamicin treatment relative to the initial inoculum. Relative percent invasions of mutant and parental strains were 0.223% ± 0.025 and 1.70% ± 0.185, respectively. Each bar represents the statistical mean of triplicated assays; error bars indicate the standard deviation (SD). Values are presented as mean ± SD.

The effect of NTG treatment–induced genetic mutations on the invasiveness of the ts mutant was assessed using an LMH cell invasion assay (Figure 3). Compared with the parental strain, the ts mutant strain 2S-G10 exhibited a lower invasion rate than that of the parental strain (*p* = 0.08).

### 3.5. Complete Genome Sequencing and Comparative Genome Analysis

**Figure 4:**
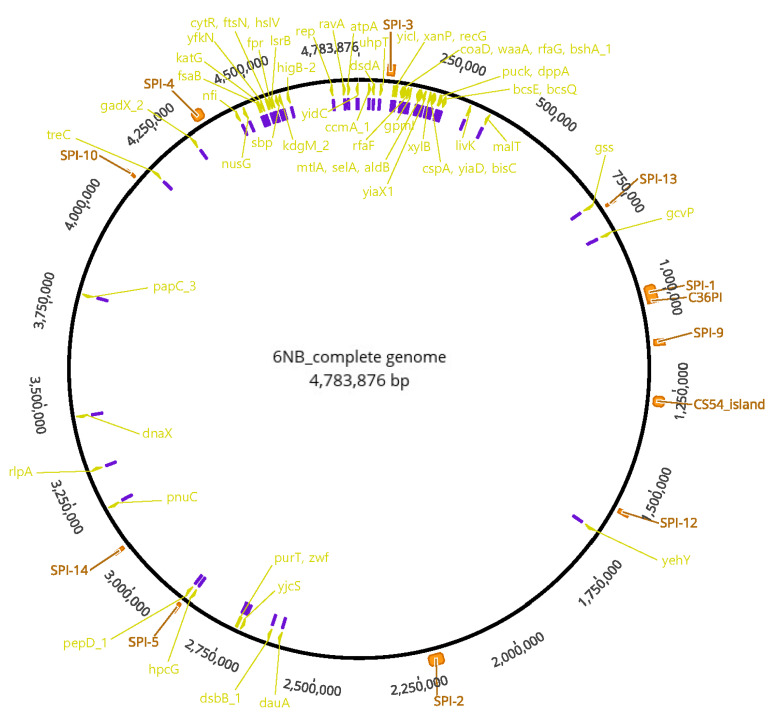
Circular genome of parental strain 6NB. The outer scale represents the size in terms of base pairs. The virulence-related genes with SNPs that occurred in the 2S-G10 strain are labeled in yellow. The SNPs related to these genes are indicated in purple. The outermost arch in orange represents the location of predicted SPIs.

We performed complete genome sequencing of the ts mutant strain 2S-G10 and its parental strain 6NB. The genome size of the parental strain 6NB was 4,783,876 bp with 52% of GC content. It comprised 4494 coding genes and a single plasmid with 75 genes. We mapped the Illumina sequencing result of the 2S-G10 strain using the 6NB complete genome sequence as a reference, thus performing a genome-wide comparison to identify the NTG treatment–induced SNPs. Of the total of 243 SNPs in the coding regions (Table S1, Supplementary Materials), 86 SNPs (35.4%) were synonymous variants, encoding the same amino acid; 131 SNPs (53.9%) were missense variants, encoding a different amino acid; and the remaining 26 SNPs (10.7%) contained frameshift variants, stop-gained variants, upstream gene variants, and conservative in-frame deletions. Among the non-synonymous SNPs, those reported in other *Salmonella* strains were excluded using MegaBLAST [24]; SNPs that did not change the structure of amino acids were also excluded. As a result, 63 SNPs located in 59 genes were predicted to be associated with the attenuation of the 2S-G10 strain, as shown in Table S2. Figure 4 shows the circular genome of the parental strain with annotated SPIs and the 63 SNPs. Notably, proline-to-serine missense mutations have been observed in *bcsE*, *recG*, and *rfaF* genes, which contribute to *Salmonella* virulence by mediating epithelial cell invasion and persistence in host systems, growth, and lipopolysaccharide core biosynthesis, respectively [25,26]. Moreover, a proline-to-serine missense mutation is present in the *pepD*_1 gene, which is located in the SPI-5 cluster.

## 4. Discussion

*Salmonella* infection in young chickens results in the rapid multiplication and spread of pathogens [27]. Immunization with various vaccines has been extensively used to prevent *Salmonella* infections in chickens. However, the available live *Salmonella* vaccines for chickens have a few safety concerns, such as the ability of the live vaccine strain to revert to its virulent form [28,29]. Moreover, live vaccine strains can interfere with *Salmonella* monitoring programs [30]. Therefore, live *Salmonella* vaccines should be sufficiently attenuated and protective. NTG is a chemical mutagen that induces a wide spectrum of mutations through the methylation of nucleic acids [9,10]. Gee et al. induced NTG-mediated mutagenesis in *Salmonella typhimurium* tester strains to determine the specificity of reversion via base substitutions and found that these strains have a low spontaneous frequency of reversion [10]. In the present study, the ts mutant strain 2S-G10 was highly attenuated in comparison with the parental strain 6NB. Indeed, the 2S-G10 strain was completely safe, as its re-isolation rates of *S*. Enteritidis strains in the liver, cecum, and cecal tonsil of infected 1-day-old chicks were negligible, unlike those of the 6NB strain, which were significantly higher than the control PBS group.

We further investigated the phenotypic features of the mutant and parental strains to assess factors associated with the loss of virulence of the ts mutant. To colonize and survive in the host poultry, *Salmonella* must survive at the high body temperature of chickens [31]. Therefore, temperature is one of the most important environmental factors that alter the expression of virulence in *Salmonella*. If the cell size is expected to be regular during the growth of the culture, OD measurements are suitable to evaluate microbial growth when OD is calibrated against the bacterial number in advance [32]. Thus, we confirmed that the number of CFUs was constant when the OD_600_ values of the two strains were the same before performing the one-step growth curve analysis (data not shown). The growth of the 2S-G10 strain was notably suppressed at 42 °C compared to that at 33 °C. This implied that the mutant strain could not colonize the chickens and was more attenuated than the parental strain, which was consistent with the result of the attenuation test in 1-day-old chicks in this study. In addition, we previously reported that this safety advantage does not reduce the protective efficacy [12]. *Salmonella* invasion of host cells is critical for the survival and establishment of infection in the host. The invasive response is initiated during the log phase, and *Salmonella* is considered maximally invasive during the late log phase [33,34,35]. However, the 2S-G10 strain showed a decreased relative invasion efficiency compared to that of its parental strain, even during the log phase. Hence, our findings indicate that 2S-G10 loses its ability to invade chicken liver epithelial cells to a certain degree.

The genomic SNP analysis between the parental strain 6NB and ts mutant strain 2S-G10 revealed a total of 243 SNPs in the coding regions comprising 86 synonymous variants, 131 missense variants, and 26 frameshift variants, stop-gained variants, upstream gene variants, and conservative in-frame deletions. While synonymous mutations may cause functional changes in rare cases [36], non-synonymous mutations, such as missense SNPs and stop-gained variants, which affect gene expression or structure, are the most likely causes of phenotypic changes. We detected 62 missense SNPs and 1 stop-gained variant among the 243 SNPs based on various criteria.

Among the protein mutations, the proline-to-serine mutation is important. Proline plays an important role in protein synthesis and structure by facilitating the folding of many proteins [37,38]. As misfolded proteins do not function properly [39], a mutation in the proline-encoding region of the virulence gene would most likely cause the attenuation. Among the genes harboring SNPs resulting in proline-to-serine changes, we hypothesized the most likely cause of attenuation to be mutations in *bcsE*, *pepD*_1, *recG*, and *rfaF* genes because in vitro experiments in this study confirmed attenuated mutant phenotypes for these genes. The *BcsE* gene is known to play several roles related to *Salmonella* virulence, such as biofilm production, biofilm morphotype, mobility, cellulose and cellulase enzyme production, stimulation of host cell immune response, epithelial cell invasion, and persistence in host systems [25]. A mutation in this gene might affect the colonization ability of the 2S-G10 strain. Missense mutations were detected in the *pepD*_1 gene, which encodes a chaperone protein. Chaperones are involved in the conformational folding and unfolding of proteins. The temperature-induced expression of chaperones contributes to cellular survival under heat stress [40,41]. Mutations in the *pepD*_1 gene of *Mycobacterium* have been shown to contribute to cellular stress response mediated through MprAB and SigE [42]. SPIs are large gene cassettes within the *Salmonella* chromosome that encode determinants responsible for establishing specific interactions with the host and expression of virulence [43]. Among SPIs, SPI-1 promotes *Salmonella* invasion into epithelial cells [44], and SPI-5 collaborates with SPI-1 [45]. Since the *pepD*_1 gene is located in the SPI-5 cluster, we predicted that this mutation would affect the invasion ability of the 2S-G10 strain. Mutation in *recG* of *Deinococcus radiodurans* caused growth defects and reduced radio-resistance [46]. This suggests that the slow growth of the 2S-G10 strain might be due to the mutation in *recG*. Van Immerseel et al. proposed that the mutation in *rfaJ* is one of the most likely causes of SG9R attenuation since an attenuated phenotype of mutant was confirmed in an animal model [47]. Our present study revealed the presence of missense mutations in *rfaF* and *rfaG*, which are clustered with *rfaJ*. All three genes encode lipopolysaccharide core biosynthesis proteins. This suggests that the slower growth of the 2S-G10 strain might be due to the defect in outer membrane formation.

Moreover, we identified nine additional genes with proline-to-serine amino acid changes. SNP analysis revealed mutations in genes involved in transport and metabolism, such as *dauA* and *gcvP*; cell motility, such as *papC*_3; energy production, such as *atpA*; replication, such as *dnaX*; signal transduction, such as *lsrB*; and transcription, such as *cytR* (Table S2). These and the remaining 39 genes are not located in the SPIs; however, they are known to affect bacterial virulence. Thus, the attenuation of the 2S-G10 strain could be potentially caused by any of the SNPs identified in this study.

Conclusively, the 2S-G10 strain showed lower virulence than the parental strain in the safety test, growth analysis, and invasion assay. We deduced that mutations in *bcsE*, *pepD*_1, *recG*, and *rfaF* genes are the most likely causes of this attenuation because these genes are associated with the phenotypes of the 2S-G10 strain. We previously proposed the ts mutant strain as a potential vaccine candidate because its oral-intramuscular immunization reduced *Salmonella* titers in the chicken spleen after infection challenge and induced strong humoral and cellular immune responses [12]. Thus, the ts mutant strain can serve as an effective vaccine against *Salmonella* infection in chickens, given an optimized immunization strategy via the oral-intramuscular route. Taken together with the present study findings, the 2S-G10 strain holds promise as a viable candidate for the generation of a novel live-attenuated vaccine against *S*. Enteritidis.

## 5. Conclusions

In this study, we describe the construction and selection of an *S*. Enteritidis ts mutant, 2S-G10, of which its immune responses and protective efficacy were evaluated in our laboratory, and presented its attenuation-related characteristics. The phenotypic features of the 2S-G10 strain are consistent with the genetic changes responsible for attenuation. Our findings suggest that the 2S-G10 strain is a potential candidate for the development of a novel live-attenuated vaccine against *S*. Enteritidis.

## Figures and Tables

**Table 1:** Oligonucleotide sequences for *Salmonella* spp. and *S.* Enteritidis specific PCR analysis.

Target (Target Gene)	Primer	5′-Sequence-3′	Amplified Product Size (bp)	Reference
*Salmonella* spp. (*sdiA*)	SdiA1	AAT ATC GCT TCG TAC CAC	274	[14]
	SdiA2	GTA GGT AAA CGA GGA GCA G		
*Salmonella* Enteritidis (*sefb*)	Sef.B127L	AGA TTG GGC ACT ACA CGT GT	535	[15]
	SefB661R	TGT ACT CCA CCA GGT AAT TG		

**Table 2:** Re-isolation of *S*. Enteritidis from the internal organs of chicks 1 week after inoculation.

Group	Type	Inoculation Dose (CFU/mL)	Number of Chicks	Liver n ^1^	Cecum/Cecal Tonsil n ^1^
PBS	Negative control	-	14	0	0
6NB	Wild-type	1 × 10^7^	12	10 ***	10 ***
2S-G10	Ts mutant	1 × 10^7^	12	0	0

^1^ n, Number of chicks with *S*. Enteritidis-positive organ. *** *p* < 0.001.

## Data Availability

The datasets generated and/or analyzed during the current study are available from the corresponding author upon reasonable request.

## Supplementary Materials

The following supporting information can be downloaded at https://www.mdpi.com/article/10.3390/vetsci10050313/s1: Table S1, SNPs detected in the *S*. Enteritidis ts mutant 2S-G10 compared with the parental strain 6NB; Table S2, The 63 SNPs identified in *S*. Enteritidis ts mutant 2S-G10 that most likely contributed to the attenuation.
